# Towards a Futureproof Zoo

**DOI:** 10.3390/ani13060998

**Published:** 2023-03-09

**Authors:** Jozef Keulartz

**Affiliations:** 1 Wageningen School of Social Sciences (WASS), Wageningen University and Research, 6700 HB Wageningen, The Netherlands; jozef.keulartz@wur.nl; 2 Institute for Science in Society, Radboud University, 6500 HC Nijmegen, The Netherlands

**Keywords:** future of the zoo, enrichment programs, training technics, more space for endangered species, broader conservationist role for zoos

## Abstract

**Simple Summary:**

Today, the animal world is under increasing pressure given the magnitude of anthropogenic environmental stress, especially climate change and habitat fragmentation. As a result of these global environmental changes, animals are becoming increasingly dependent on care in conditions of temporary or permanent captivity. Hence, today’s zoos are tasked with a growing obligation to foster and promote the conservation of threatened and endangered species. To successfully fulfil this task, the zoo must strive to find a morally acceptable balance between animal welfare concerns and species conservation commitments—a difficult challenge, especially as both wildlife conservationists and animal protectionists tend to oppose any attempts at such a value balancing.

**Abstract:**

To develop an adequate ethical framework for a futureproof zoo, we have to employ what I would call a ‘bifocal’ view, in which zoo animals are seen simultaneously as individuals in need of specific care and as members of a species in need of protection. From such a bifocal view, the zoo’s policy should aim to strike a fair, morally acceptable balance between its effort to ensure the welfare of individual animals and its obligation to contribute to species conservation. I will argue that the prospects of the zoo to achieve such a balance are promising. Since early 21st century, zoos have made serious and sustained efforts to ensure and enhance animal welfare. The zoo’s huge animal welfare concerns are reflected in the development of animal enrichment programs and the increased use of training technics. At the same time, the zoo’s contribution to species conservation has also improved considerably. Zoos have found solutions for the problems created by their lack of space, such as innovative enclosure designs, specialization, regional and global cooperation, the interactive exchange of in situ and ex situ populations, and the shift away from large charismatic mammals towards smaller species. Zoos have also improved their conservation performance by broadening their conservationist role to include research, training, education, awareness campaigns, and direct financial and technical support for in situ projects. I will occasionally illustrate certain developments using examples drawn from ARTIS Zoo, the fifth oldest zoo in the world, located in the centre of my hometown Amsterdam.

## 1. Introduction

Embarking on the quest for a proper ethical framework, it is essential to realize that zoos are currently in the process of transition from venues of outdoor public education and entertainment to fully fledged conservation centers.

The opening of the Zoological Garden in Regents Park in 1828, also known as the London Zoo, marked the birth of the modern zoo. It was the first zoological garden that was explicitly presented as the center of scientific study and zoological education. However, the mission of the founding generation to focus only on science and education, and consequently, to disassociate themselves from the traveling menageries and circuses of the time, that offered little more than entertainment, failed. By the second half of the nineteenth century, zoos came under increasing financial pressure and had to open their gates to the general public to secure their survival—hence the zoo’s new motto: ‘education and entertainment’.

That changed in the 1970s and 1980s when zoos began to turn their attention to the conservation of endangered species and wildlife. A major milestone in this development was the Convention of Biodiversity, which was signed at the Earth Summit in Rio de Janeiro in 1992. Article 9 of that convention deals explicitly with the role of ‘ex situ conservation’: nature management outside the natural habitat of animals, particularly in seed banks, arboreta, botanical gardens, zoos, and aquariums.

In 1993, in the wake of this world summit, the World Association of Zoos and Aquaria (WAZA), in close cooperation with the International Union for Conservation of Nature (IUCN), launched the first World Zoo Conservation Strategy—under the motto: ‘Captivity for Conservation’. Its conclusion explicitly stated that, at a time when species, habitats, and ecosystems worldwide are threatened with extinction, modern zoos must commit to the conservation of species and wildlife. “Caring for our planet’s biological systems is one of the greatest challenges to humankind. Consequently, conservation is being seen as the central theme of zoos, and zoos should thus further evolve into conservation centres”.

Another important milestone concerns the so-called One Plan Approach, launched in 2012 at the IUCN World Conservation Congress. According to this plan, in situ populations (in the wild) and ex situ populations (in captivity) must be managed as one coherent whole. This management approach, that is also referred to as inter situ conservation [1] or pan situ conservation [2], mainly involves the mutual exchange of both populations. Zoo animals can be used for restocking declining populations or for reintroducing locally extinct populations, while supplying animals from wildlife populations can help improve the genetic and demographic viability of zoo populations [3].

With the One Plan Approach, the distinction between classic in situ and ex situ conservation is becoming blurred to the point of disappearing entirely. We witness what Irus Braverman [4] (p. 15) has called a shift ‘from bifurcation to amalgamation’ of in situ and ex situ conservation. The dissolution of the boundaries between the ‘wild’ and the ‘walled’ and the integration of zoo-based tools and techniques in species conservation has caused wildlife parks and nature reserves to become increasingly similar to zoos, and vice versa, leading to numerous clashes between animal rights activists and wildlife conservationists.

Both parties adhere to opposing ethical frameworks. Animal ethicists use an individualistic framework in which species are of little or no moral relevance. According to Tom Regan, who developed an influential theory of animal rights, species cannot claim any rights, including the right to survive. The rights view’s answer to the question whether zoos are morally defensible, “not surprisingly, is No, they are not” [5] (p. 46). Another animal ethicist who had at least as much influence on the animal rights movement as Tom Regan is Peter Singer. In his book Animal Liberation from 1975, Singer argues against what he calls ‘speciesism’, the belief that humans are infinitely more valuable than non-human animals. According to Singer, this is a prejudice that is akin to the prejudice that sexists have in favor of their own gender, and that racists have in favor of their own race. Not surprisingly, this idea is reflected in the work of ecofeminists, posthumanists, and anticolonialists [6,7,8].

Animal ethicists of all sorts agree with Regan and Singer that only individual animals have intrinsic value and moral standing, not collective entities, such as species or ecosystems. Environmental ethicists, on the other hand, generally use a holistic framework, in which the rights and welfare of individual animals are subordinate to the interests of the larger whole of which they are a part, such as species and ecosystems. Michael Hutchins, who held a leading position in the Association of Zoos and Aquaria (AZA) and in The Wildlife Society (TWS), has argued that animal rights and conservation ethics are inherently incompatible, and “that one cannot be an animal rights proponent and a conservationist simultaneously” [9] (p. 816). 

Putting an end to the seemingly intractable controversies between animal protectionists and wildlife managers, and integrating their conflicting ethical frameworks will require the development of a bifocal view in which zoo animals are seen simultaneously as individuals in need of specific care and as members of a species in need of protection [10]. It could even be argued that animal welfare and wildlife management are two sides of the same coin, “by showing that the integrity of habitats and the populations they contain are inextricably linked to the welfare of the individual animals that constitute those populations and occupy those habitats” [11] (p. 179). If animal welfare concerns are ignored, wildlife conservation policies are likely to fail, and vice versa [12] (p. 123).

From a bifocal view of zoo animals as individuals and as members of a species, zoos will pursue ethical policies only if they manage to strike a fair balance between animal welfare considerations and species protection obligations. The prospects for zoos to achieve such a balance are promising. In the past decades, zoos have been striving to ensure and improve animal welfare, and making significant progress in species conservation. 

## 2. Zoo Animal Welfare

Since the opening of Carl Hagenbeck’s zoo in 1907, the taxonomic group has given way to the animals’ natural habitat in the design of animal enclosures. However, this process of naturalization obviously runs up against limits. Real habitats take up a lot of space—just think of the area required by tigers. Moreover, some forms of predator behavior, such as hunting and killing prey animals, cannot be realistically simulated in captivity. Additionally, of course, the same is true of prey animal behavior. Captivity thus deprives wild animals of the necessity and opportunity to perform those tasks important to their survival, such as finding food and evading enemies, a situation that inevitably leads to boredom. The view that captivity necessarily entails a deprivation of animal welfare is characteristic of the perspective in which this welfare depends on the extent to which animals can exhibit species-specific behavior. This is not the case if one adopts the perspective that the welfare of animals depends on their ability to adapt without suffering to the environments provided by man [13].

To overcome the problem of boredom, we need to turn to ‘occupational therapy’, according to Heine Hediger, known as the ‘father of zoo biology’. “The captive animal must be given a new interest in life, an adequate substitute for the chief occupations of freedom” [14] (p. 158). In Frontiers of Justice, the philosopher Martha Nussbaum cites an example of such a substitution [15] (p. 371). This involves the ability of tigers in zoos to exercise their predatory behavior without actually killing prey animals. At Bronx Zoo, to the tigers’ satisfaction, they have replaced the gazelle with a large ball on a rope that imitates the weight and resistance of this prey animal.

### Enrichment Programs

The introduction of such objects—‘toys and treats’—is just one of many options for providing what is known as behavioral or environmental enrichment. This form of animal care aims to encourage the exercise and development of species-specific behaviors and skills, thereby improving animal welfare. In addition to toys and treats, the way food is made available also provides opportunities for enrichment. Three strategies for food enrichment can be distinguished: increasing the number of daily feeding sessions; making the feeding schedule less predictable; and making the food less easy to obtain, either by hiding it or providing live prey or whole carcass meals.

This latter strategy of food enrichment is not uncontroversial. When in the 1970s Woodland Park Zoo began feeding whole dead sheep and goats to the big felines, and whole dead rabbits and chickens to the small felines, many visitors showed bewilderment, repulsed by the sight of meat being torn off recognizable carcasses. By now, people seem to have gotten used to it. However, feeding live animals is probably still a bridge too far.

Another important enrichment strategy concerns the improvement of animal enclosure design by creating a network of corridors and paths, especially between the treetops, that allow the animals to rotate between different enclosures [16]. The idea of such a rotation system is based on Hediger’s theory of wildlife territory [14], consisting of a multitude of separate places connected by paths where it is possible to alternate between foraging, sleeping, relaxing, et cetera.

Other enrichment strategies include sensory enrichment in the form of auditory, olfactory, and visual stimuli, and social enrichment, which allows animals to interact with other animals, either with animals of the same species or with animals of other species. A good example of the latter are mixed-species enclosures. They provide an exciting experience for the animals, visitors, and for zoo staff. In such enclosures, activity levels tend to be high, with many play behaviors, and this has a positive effect on the physical and mental health of the animals [17]. Moreover, interactions with animals of different species can contribute to successful reintroduction. Animals from mixed-species enclosures usually cope better with the complexity of their natural habitat to which they will be released.

Finally, training is also an important form of enrichment. Training can provide animals with the motivation, skills, and confidence needed to make the most successful use of the enrichment facilities provided. Through training by positive reinforcement, animals can also learn to participate in their daily health and husbandry care without sedation or restraint. By voluntarily cooperating during dental procedures, blood draws, urine collections, stethoscope examinations, artificial inseminations et cetera, they can benefit from the preventive and curative health benefits of veterinary treatments. This can also significantly reduce the stress usually associated with these operations. Furthermore, training is also important for the relationship between the animals and their caretakers. Zoo animals are dependent on humans, and their well-being will increase as a bond of trust with caregivers develops, a bond that can be significantly strengthened through training.

## 3. Species Conservation

Since the World Zoo Conservation Strategy from 1993, the zoo has been considered a Noah’s Ark, which owes its raison d’être mainly to its contribution to wildlife conservation through breeding and reintroduction programs. However, the Ark soon sustained severe damage when these programs proved to run into major problems. 

Zoos suffer from a severe lack of space and actually have room for only a small number of endangered species. That problem is reinforced since many of the animals that are currently living in zoos are not endangered, while zoos are generally very reluctant to give up popular animals that are not threatened with extinction. A study published in 2011 showed that only 15 percent of terrestrial vertebrates held in captivity are threatened or endangered [18].

To make matters worse, zoos struggle to successfully breed even these few species because the populations are usually too small. Originally, breeding programs aimed to maintain 90% of a species’ genetic variability over a 200-year period [19]. As such a long period requires very large numbers of animals per species, it was shortened from 200 to 100 years in the mid-1990s. However, most breeding programs lack even enough space to achieve this downwardly revised goal.

This situation often creates the problem of so-called ‘surplus’ animals. These animals are considered surplus because they do not (or no longer) fit in the zoo’s breeding program. Traditional methods to solve this problem include culling the surplus animals or selling them to dealers, where they often end up in substandard facilities. These methods are among the most sensitive PR issues facing zoos, and threaten to seriously undermine their credibility as Noah’s Ark. This became abundantly clear in a case that sparked worldwide outrage, namely the case of Marius, a healthy young giraffe, who was publicly killed at the Copenhagen Zoo in 2014, dismembered, then fed to lions. As Leslie Kaufman [20] aptly noted, breeding endangered animals in this way “feels less like Noah building an ark and more like Schindler making a list”.

Not only are the success rates of breeding programs disappointing, the success rates of reintroduction programs are also disappointing, often due to insufficient consideration of ecological, social, economic, and political aspects. To increase these success rates, zoos have made great efforts to improve the pre-release training. Pre-release training is aimed at maintaining or developing the skills that may have been lost in captivity, such as orientation and navigation, finding or building suitable nest sites, hunting and foraging behavior, and predator avoidance. 

Predator avoidance training is essential for the success of reintroduction programs, as a large amount of post-release deaths can be attributed to predators. To prepare animals for release, they are typically exposed to live predators or predator models, accompanied by aversive or stressful stimuli, such as an alarm. In the case of ‘mesopredators’—animals which are both predators and prey—antipredator training needs to be combined with the development of predatory skills. A good example of a species which requires both types of training is the black-footed ferret [21].

In 1986, the population of ferrets had diminished to a mere 18 individuals, but thanks to a captive breeding program, many hundreds of them are once again roaming the prairie of the U.S. state of Wyoming. This did not happen without difficulty. The animals suffered from prey-naivety and failed to hide from predators, such as eagles, coyotes, and badgers. Researchers developed a model predator: a stuffed badger on wheels, which would become known as RoboBadger. The ferrets could only escape RoboBadger by crawling into a burrow. The researchers then tried to increase the ferrets’ aversion to RoboBadger by firing rubber bands at them. This was, by the way, not a great success as became clear when the ferrets started riding on the back of RoboBadger. 

The ferrets must not only figure out how to avoid predators, but also how to capture and kill prairie dogs, which constitute 65–90% of their diet. Furthermore, they must discover how to occupy prairie dogs’ burrows, since they do not construct their own. During their 30-day preconditioning period, ferrets are ideally able to kill four prairie dogs and live in a real prairie dog burrow. 

Some critics of the zoo perceive a tension at this point: on the one hand, training is aimed at facilitating animals’ adaptation to a human environment, but on the other hand, some animals are being prepared for an existence in the wild, where they will have to adapt to a natural environment. The animals have to switch from domestication and human dependency to dedomestication and nature dependency. However, animals are apparently capable of making such a switch. This is certainly true for the ferrets who received a pre-release training that will increase their survival rate by a factor of ten compared to animals released directly from the cage [4] (pp. 119–123). In any case, the potential loss of animal welfare should be weighed against the possible gain in species conservation.

Given how expensive and unsuccessful the breeding and reintroduction programs have turned out, Noah’s Ark seemed headed for shipwreck at the turn of the century. To keep the Ark afloat, the zoo needed all hands on deck. As a result of its efforts, the zoo has managed to significantly improve its contribution to species conservation. Zoos have found solutions for the problems created by their lack of space, such as innovative enclosure designs, specialization, regional and global cooperation, the interactive exchange of in situ and ex situ populations, and the shift away from large charismatic mammals towards smaller species (Section 3.1). Zoos have also improved their conservation performance by broadening their conservationist role to include research, training, education, awareness campaigns, and direct financial and technical support for in situ projects (Section 3.2).

### 3.1. Creating More Space

To address the problem of the vast lack of space, zoos have a number of options. As we have already seen, they can create more space by building a network of trails that gives animals the opportunity to rotate between various interconnected display and off-display areas. They can also reduce the number of species in order to be able to build larger enclosures. To give an example: ARTIS Zoo has reduced the number of animal species since 1967 from over 1800 to around 500. 

To improve the success of breeding programs, zoos should reduce the number of species that are not endangered and specialize in those that are [22]. They should also promote and facilitate regional and global cooperation. Joint management and exchange of animals between zoos can solve the problem of low numbers, thus enhancing the demographic and genetic stability of zoo populations.

Initiatives to develop and support cooperative management of captive animal populations have been underway for some time. In the early 1980s, the North American Association of Zoos and Aquariums (AZA) launched so-called Species Survival Plans (SSPs) to facilitate cooperation in breeding programs between accredited zoos across the region. Similar cooperative programs were developed by other regional organizations, such as the European Association of Zoos and Aquariums (EAZA), and Australasia’s Zoo and Aquarium Association (ZAA). Since 2003, interregional cooperation programs have also been developed, the so-called Global Species Management Plans (GSMPs).

Another way to combat the problem of small numbers, besides the regional and interregional exchange of animals between zoos, is the integration of in situ and ex situ programs through the One Plan Approach, that opens the possibility to simultaneously improve the stability and sustainability of the wild and captive populations of endangered species. 

However, by far the most effective strategy to combat the space problem is undoubtedly to change the composition of zoo collections. The long-standing predominance of charismatic flagship species—lions, tigers, giraffes, elephants, bears, hippos, and rhinos—does not reflect the diversity of animal species in the wild. Zoos need to shift away from such large mammals towards smaller species, especially amphibians, invertebrates, and some fish species. These species not only take up less space, but are also relatively inexpensive to keep, have fewer welfare problems in captivity, have high birth rates, and are easy to reintroduce [23]. 

Some fear that such a shift to smaller species will come at the expense of the zoo’s attraction to visitors—after all, who would be interested in a Praying Mantis? Zoos need to balance conservation credibility with commercial viability; to reach the aim of species conservation they need to attract visitors. However, the assumption that zoos will not attract enough visitors without large mega-vertebrates is far from uncontroversial. Research findings show that while large charismatic animals, such as tigers, elephants, and great apes, perform an important role in attracting people to the zoo, once they are inside, smaller species can easily rival these large animals in terms of popularity and attention. They also suggest that imaginative displays of small-bodied species can substantially increase zoo attendance [24] (p. 79). 

Once again, this can be illustrated with an example from ARTIS Zoo, where, in September 2014, the Dutch queen Máxima opened the first museum of micro-organisms Micropia. The museum uses 3D viewers to show visitors how microbes move, eat, and reproduce, and interactive displays, such as a body scanner that reveals the types of microbes living on the body, and a Kiss-o-meter which counts the number of microbes transferred during a kiss [23] (p. 347).

### 3.2. Broadening the Zoo’s Conservationist Role

By the turn of the century, the vision of the zoo as Noah’s Ark has been given way to a much broader approach. This shift also becomes apparent when comparing the first 1993 World Zoo Conservation Strategy with the new 2005 World Zoo and Aquarium Conservation Strategy, entitled ‘Building a Future for Wildlife’. The first document explicitly describes reintroduction as the ultimate goal of ex situ conservation. The second document recognizes reintroduction as a useful tool for species and wildlife conservation, but cautions against excessive expectations because returning animals to the wild is a difficult and long process. This document outlines an ‘integrated approach’, in which all major zoo activities, including financial support and fundraising, research and training, and education and awareness campaigns, serve the protection of endangered species and the conservation of healthy ecosystems [25,26,27].

The simplest way zoos can contribute to in situ conservation is through financing and fundraising. With about $350 million annually, WAZA member zoos are the third largest financial supporter of species and wildlife conservation after the Nature Conservancy and the World Wildlife Fund [18]. Since 2015, WAZA zoos have been stepping up their efforts. That year, the next World Zoo and Aquarium Conservation Strategy was published, entitled ‘Committing to Conservation’. In this report it is argued that every zoo should devote at least 3% of their budgets to conservation work. If all WAZA institutions will take up this challenge, they shall surely become the largest funder of in situ conservation.

Zoos can employ various fundraising options. Visitors can make a financial contribution ‘on the spot’ to a conservation project of their choice. They can make use of conservation contribution machines to donate cash towards the conservation of certain species. However, much more lucratively: zoos can invite companies to sponsor in situ conservation programs or to adopt an animal or animal enclosure—at ARTIS Zoo, one can adopt an animal from EUR 2500 annually. In return for their philanthropy, companies will enjoy benefits, such as joint public relations activities, and the mention of brand names in the zoo and online. 

Fundraising proceeds are typically used to support various organizations working to protect the natural habitats of animal species, thus preventing wild populations from extinction. ARTIS Zoo supports, among others, the Fundacion Rewilding Argentina, which is committed to restoring the Iberá Natural Reserve, the second largest wetland in the world, and the home of many endangered species that also live in ARTIS Zoo, such as the giant anteater, the green-winged macaw, the South American tapir, and the jaguar.

With the support of ARTIS Zoo, GPS collars were purchased to track jaguars. Thanks to the collars, the project workers are able to monitor the animals and the locals are aware when a jaguar is nearby, allowing them to move their livestock to a safer location, reducing the threat of the jaguar. Additionally, the support from ARTIS Zoo is used for feeding young giant anteaters that are taken in from the area, and later, when they are fully grown, for a GPS harness to track them.

Since 2018, ARTIS Zoo has also been supporting Operation Jaguar in its mission to prevent poaching and illegal trading of jaguars in countries, such as Bolivia, Suriname, and Guyana. The demand for jaguar body parts, such as teeth and bones, primarily comes from Asia, and traffickers often use air travel to transport their contraband. To combat this, airports are utilizing dogs that are trained to sniff out the scent of jaguars. ARTIS Zoo has been helping in this endeavor by providing blankets that have been lying around its jaguars and their poop and urine to make the dogs familiar with the smell of the feline.

In addition to financing and fundraising, zoos also have an important role in supporting in situ conservation through research and training. Exporting expertise is at least as important as repatriating animals [28] (p. 169). Studies of animal behavior, genetics, reproduction, and nutrition conducted within zoos are becoming increasingly relevant for in situ conservation as natural habitats continue to be damaged and destroyed at the current rate.

As the size and genetic diversity of wildlife populations is progressively declining, these populations are becoming similar to zoo populations. The expertise of zoos with small, but genetically and demographically healthy populations of captive animals has proven useful in maintaining small and shrinking populations in the wild. Zoo expertise also performs an important role in almost all stages of the exchange process between wild and captive animals, from capture, transport, breeding, and pre-release training, to the (re)introduction of endangered or locally extinct animals. Moreover, zoos can support in situ conservation by providing technical skills and by training conservation scientists [29] (p. 282).

However, the perhaps most important contribution of zoos to in situ conservation comes from education, awareness, and advocacy. It is realized by now that the 700 million people that annually visit zoos are significant conservation assets, at least as valuable as the zoos’ animals [30]. In North-America, more people visit zoos and aquariums than all major sports (baseball, basketball, hockey and football) combined. In 2019, the year before the outbreak of the corona pandemic, ARTIS Zoo received over 1.4 million visitors, which is more than all other top locations in Amsterdam.

Only recently zoos have started to conduct research into their educational impact on the knowledge, awareness, and behavior of visitors, in order to better fulfill their role as catalysts of change. It would take some time for research on zoo visitors to reach maturity. An early large study, which for the first time found direct evidence that zoos have a positive impact in changing visitors’ feelings and attitudes about conservation [31], was heavily criticized for containing “at least six major threats to methodological validity that undermine the author’s conclusions” [32] (p. 126). 

However, a number of methodologically robust studies are now available that convincingly demonstrate the potential educational value of zoo visits. One such study was conducted by Eric Jensen [33]. His large-scale study (n = 2839), which is focused on the educational impact of zoo visits on children and adolescents, lacks the methodological shortcomings of previous studies, and broadly supports the idea that zoo visits can have a positive effect on learning processes and attitude changes towards nature and conservation. To enhance that effect, ARTIS Zoo organizes guided tours, lecture series, workshops and courses for children, youth, and adults. Additionally, there are 300 volunteers who are always available at the enclosures to give explanations and answer questions.

In order to further increase the educational effectiveness of zoo visits to raise awareness and encourage willingness to act to the benefit of nature conservation, zoos have shifted their focus from cognitive to affective learning. Furthermore, they have recognized that behavioral changes can only be achieved on some measurable scale when zoos can inspire people to take low-threshold and achievable nature- and animal-friendly actions [34] (p. 140).

An example of this is the Seafood Watch, a fish guide that is handed out to visitors of the Monterey Bay Aquarium. It has been shown that this fish guide has significantly influenced the buying behavior of 80% of its visitors. Another great example is the They’re Calling on You campaign of the Melbourne Zoo. Visitors to the gorilla enclosure are asked to donate their old mobile phone. The phones are then offered to a recycling company. This not only helps reduce the environmental impact of coltan mining, which threatens gorilla habitats, but also raises funds for gorilla conservation.

## 4. Concluding Remarks

As species, habitats, and ecosystems around the world face the danger of extinction, the borderline between in situ and ex situ conservation has become blurred to the point of disappearing entirely, and the use of zoo-based tools and techniques are increasingly being adopted in wildlife management. Under these circumstances, there is urgent need to develop a bifocal view that allows for seeking and exploring common ground between animal protectionists and wildlife conservationists, to transcend their differences and move beyond the black-and-white thinking that has led their debates time and again to a total deadlock. On the basis of present trends—the development of animal enrichment and training programs on the one hand and the improvement of the contribution to in situ conservation on the other—one can conclude with some confidence that the prospects for zoos to achieve an ethically acceptable balance between animal welfare concerns and species conservation commitments look rather promising.

## Data Availability

Not applicable.
